# Beta‐cell function in type 2 diabetes (T2DM): Can it be preserved or enhanced?

**DOI:** 10.1111/1753-0407.13446

**Published:** 2023-07-31

**Authors:** Laure Sayyed Kassem, Aman Rajpal, Margarita Victoria Barreiro, Faramarz Ismail‐Beigi

**Affiliations:** ^1^ Case Western Reserve University Cleveland Ohio USA; ^2^ Cleveland VA Medical Center Case Western Reserve University Cleveland Ohio USA; ^3^ University Hospitals of Cleveland Cleveland Ohio USA

**Keywords:** beta‐cell mass, beta‐cell replication, beta‐cell survival, disposition index, insulin resistance, 胰岛素抵抗, β细胞数量, β细胞复制, β细胞存活, 处置指数

## Abstract

Type 2 diabetes (T2DM) is a complex metabolic disorder manifested by hyperglycemia, insulin resistance, and deteriorating beta‐cell function. A way to prevent progression of the disease might be to enhance beta‐cell function and insulin secretion. However, most previous studies examined beta‐cell function while patients were using glycemia‐lowering agents without an adequate period off medications (washout). In the present review we focus on studies with a washout period. We performed a literature search (2010 to June 2021) using beta‐cell function and enhancement. The evidence shows that beta‐cell function can be enhanced. Bariatric surgery and very low calorie diets show improvement in beta‐cell function in many individuals. In addition, use of glucagon‐like peptide‐1 receptor agonists for prolonged periods (3 years or more) can also lead to improvement of beta‐cell function. Further research is needed to understand the mechanisms leading to improved beta‐cell function and identify agents that could enhance beta‐cell function in patients with T2DM.

## Graphical abstract

Highlights

- Type 2 diabetes mellitus (T2DM) is characterized by progressive loss of beta‐cell function and mass. Numerous mechanisms of beta‐cell failure are involved.
- Preservation and/or enhancement of beta‐cell function is of great importance in delaying the progression of T2DM and the burden of its complications.
- Accurate evaluation of the effects of T2DM therapies on beta‐cell function require assessments of beta‐cell function following a washout period. This review focuses primarily on such assessments.
- Of all available therapies for T2DM, significant and sustained weight loss as well as prolonged therapy with glucagon‐like peptide‐1 receptor agonists are associated with preservation and perhaps enhancement of beta‐cell function.

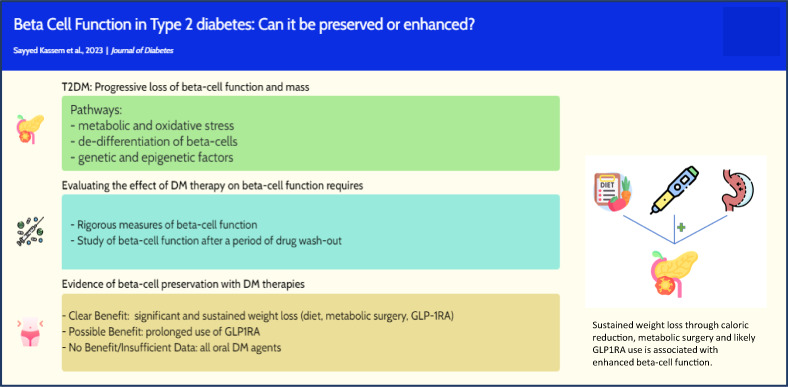

## 摘要

2型糖尿病(T2DM)是一种复杂的代谢紊乱，表现为高血糖、胰岛素抵抗和β细胞功能逐渐恶化。预防该疾病进展的一种方法是增强β细胞功能和胰岛素分泌。然而，此前大多数研究在患者使用降糖药物且没有足够的时间停药(洗脱期)的情况下检查了β细胞功能。在本文中，我们重点讨论了有洗脱期的研究。我们使用了β细胞功能和增强作为关键词进行了文献检索(2010年至2021年6月)。现有证据表明，β细胞功能可以增强。减重手术和极低热量饮食可改善许多个体的β细胞功能。此外，使用GLP‐1受体激动剂进行长期治疗(3年或更长时间)也可以改善β细胞功能。需要进一步的研究来了解导致改善β细胞功能的机制，并确定可以增强T2DM患者β细胞功能的药物。

## 1 INTRODUCTION

Type 2 diabetes (T2DM) is a complex and not fully understood metabolic disorder with genetic and familial predispositions for developing the condition. Patients manifest hyperglycemia, often are overweight or obese, have insulin resistance, hypertension and dyslipidemia, and a heightened level of inflammation.^1^, ^2^ The hyperglycemic state is mediated by increased production of glucose by the liver and kidneys, excessive renal reabsorption of glucose, rapid emptying of stomach, and increased appetite and food intake.^3^, ^4^ These changes occur in the setting of increased resistance to insulin action, which necessitates higher secretion and blood levels of insulin to maintain proper glucose homeostasis.^5^, ^6^

Studies on the natural history of T2DM have documented that hyperglycemia develops once insulin secretion is no longer adequate for the metabolic state.^7^, ^8^ Control of hyperglycemia in patients with T2DM helps prevent the onset and progression of microvascular complications (retinopathy, nephropathy, and neuropathy) and perhaps macrovascular disease.^9^, ^10^, ^11^, ^12^

An important way to prevent and/or delay the onset of hyperglycemia might be to preserve and/or enhance beta‐cell number and function for a significant period of time (years). Increasing evidence for this hypothesis is becoming available. In keeping with this, it is highly likely that improved function of beta‐cells (and hence insulin secretion) will delay the onset of hyperglycemia and reduce complications.

Previous studies have focused on interventions that preserve beta‐cell function, and we and others have reviewed this progress.^13^, ^14^, ^15^ However, most of those studies examined beta‐cell function while the individual was still undergoing treatment with glycemia‐lowering agents without an adequate “washout” period (off of the therapeutic agent). Preservation has been defined as no diminution of beta‐cell function over time. However, given the slow rate of decline in beta‐cell function in T2DM (5%–10% per year),^16^, ^17^ it is often not clear whether a small change of beta‐cell function over a short study period has validity.

During the past decade more studies have documented improvement of beta‐cell function following specified therapies. Here we define *enhancement* (or *improvement*) as having higher beta‐cell function off of therapy with the therapeutic agent (after a reasonable washout period) compared to beta‐cell function measured at the initiation of the study. This definition is in keeping with the recent definition of *remission* of T2DM by several associations (ie, normal glycemia after 3 months of discontinuation of glycemia‐lowering medications, lifestyle changes, or bariatric surgery) that is most probably due to improved beta‐cell function.^18^ It is worth emphasis that correction of hyperglycemia addresses only a part of the metabolic derangements in patients with T2DM and improvements in other T2DM‐related abnormalities are necessary for complete remission of the disease.

In this review, we present a brief overview of beta‐cell physiology and pathophysiology, then we focus only on studies that have documented either preservation and/or enhancement of beta‐cell function that included a “washout” period. Whereas clinically positive effects of medications on beta‐cell function while on treatment are highly desirable, durable effects (disease modifying) can be assessed only when the medication has been withdrawn.

## 2 METHODS

A MEDLINE PubMed search was performed from 2010 to June 2021 using the terms “beta‐cell function” and “beta‐cell preservation” in combination with various therapeutic interventions. Our prior review of this topic included data through 2009.^13^ Only trials with rigorous measures of beta‐cell function including disposition index (DI) (or equivalent measures) *plus* a washout period were selected for inclusion. “Acceptable” washout periods were determined by drug half‐life: >24 h for oral antihyperglycemic agents, >2 months for thiazolidenediones, >48 h for daily glucagon‐like peptide (GLP‐1) receptor agonists (RAs), and >8 weeks for weekly GLP‐1 RAs. Trials with less rigorous measures were reviewed when deemed pertinent but were not included in the tables. Trials based on these criteria, regardless of their design (randomized control trial, uncontrolled, or randomized crossover) with positive or negative outcomes were included. Review articles, editorials, letters to the editors, commentaries, case reports and case series were not included.

### 2.1 Beta‐cell Physiology and Pathophysiology

#### 2.1.1 Beta‐cell mass from fetal stages to adulthood

Based on autopsy studies, the number of beta‐cells in humans rapidly increases in midfetal stages, and by 2 years the number of beta‐cells approach those seen in islets of young adults.^19^, ^20^, ^21^, ^22^ Nonetheless, a small number of replicating Ki 67^+^ beta‐cells (~1%) are still present in islets and pancreatic ducts into late adulthood.^21^, ^23^ Sources of beta‐cells include nonpancreatic somatic cells, ductal cells of excretory pancreas, and beta‐cells themselves. Beta‐cells have a long lifespan of up to 25–30 years.^23^

In a landmark autopsy study, Butler and collaborators reported relative beta‐cell volume (defined as the ratio of beta‐cell area/exocrine area) in several conditions.^24^ They found that relative beta‐cell volume varied significantly (~4–5 fold) in normal nondiabetic and nonobese adult individuals. This variability probably reflects the number of beta‐cells in the fetus, genetic factors, conditions of pregnancy as well as the nutritional status and body mass index of the mother.^25^ They also found that relative beta‐cell volume was significantly higher (by about 30–50%) in obese individuals compared to nonobese people. Finally, they reported reduced beta‐cell volume in both obese and nonobese individuals with T2DM who exhibited high rates of apoptosis. Many of these findings have been replicated by other investigators.^26^

Conditions that are associated with higher than normal rates of beta‐cell neogenesis/replication in humans include pregnancy, obesity, and during the early stages of type 1 diabetes.^27^, ^28^, ^29^ In the latter condition growth occurs in both alpha‐ and beta‐cells and is probably in response to inflammation and immune‐responses.^30^ There is also evidence that under conditions of stress or aging, beta‐cells can de‐differentiate into alpha cells or other types of islet cells (see further discussion later).^31^ It should be noted that at any given time only a minority of beta‐cells are active and secret insulin.^32^, ^33^

#### 2.1.2 Factors that can stimulate proliferation of beta‐cells

Whether it becomes possible to pharmacologically stimulate the proliferation and neogenesis of beta‐cells (or decrease their rate of apoptosis) in patients with T2DM is a topic of great clinical relevance. Research in the past 2 decades has highlighted the critical role of the physiological rise of intracellular calcium concentration in beta‐cell physiology.^34^ A rise in calcium stimulates insulin secretion in response to elevated blood glucose. Additionally, calcium/calmodulin complexes, through downstream factors, stimulate the rate of insulin gene transcription and synthesis, stimulate beta‐cell replication, and play an important role in beta‐cell survival. Calcium/calmodulin complex activates the phosphatase enzyme calcineurin and leads to de‐phosphorylation of the nuclear factor of activated T cells (NFAT); this results in transcriptional stimulation of several genes operative in the pathway of beta‐cell replication and survival.^34^ Of note, the immunosuppressing agents tacrolimus and cyclosporine A inhibit calcineurin and contribute to decreased beta‐cell function in patients with organ transplantation. Finally, using single cell mass cytometry and high‐throughput small molecule screens, nonglycemia‐lowering agents, including the plant alkaloid harmine and 5′iodo‐tubercidin (5‐IT), have been found to stimulate beta‐cell replication (including in human islets in vitro) through the NFAT pathway.^35^, ^36^

### 2.2 Proposed mechanisms of beta‐cell failure

Numerous mechanisms have been proposed to explain the reduction in beta‐cell mass and function in the development and progression of T2DM. Broadly, they encompass genetic and epigenetic factors,^14^ de‐differentiation of beta‐cells, increased apoptosis, metabolic and oxidative stress, and hyperglycemia itself. Principal metabolic stressors include insulin resistance, increased caloric intake, hyperglycemia, hyperlipidemia, and a heightened systemic inflammatory state.

Oxidative stress has been linked to decline in beta‐cell function. The beta‐cell is especially susceptible to oxidative stress due to its limited capacity to clear oxygen radicals. Although this provides an embryologic advantage because some oxidative stress is necessary for beta‐cell neogenesis and proliferation,^37^, ^38^ it presents a disadvantage in the setting of increased demand for proinsulin synthesis and folding that is associated with higher amounts of reactive oxygen species (ROS). Hyperglycemia also increases the mitochondrial output of ROS.^39^ Similarly, high levels of free fatty acid metabolism result in generation of excessive ROS.^40^ Glucotoxicity activates the hexosamine pathway and contributes to beta‐cell dysfunction partly by increasing oxidative stress.^41^ Activation of protein kinase C by hyperglycemia and by inflammatory states is associated with reduced beta‐cell function, and protein kinase C is a mediator of beta‐cell injury through interleukin‐1 proinflammatory pathways.^42^

Hyperlipidemia promotes insulin resistance and exerts direct stress on beta‐cells to use excess lipids. Data in individuals at risk for T2DM show a strong correlation between increasing levels of nonesterified fatty acids and declining beta‐cell function.^43^ Intracellular toxic metabolites including ceramides promote beta‐cell dysfunction by decreasing proinsulin synthesis, increasing ROS production, inducing proinflammatory cytokine responses,^44^, ^45^ and stimulating apoptosis by activating caspases.^46^ Importantly, the synergism of glucotoxicity and lipotoxicity appears to further exacerbate beta‐cell dysfunction and death.^44^, ^45^

Beta‐cells possess a highly developed endoplasmic reticulum (ER) and Golgi apparatus that enables large amounts of protein synthesis, folding, packaging, and transport. Under normal conditions, proinsulin synthesis accounts for approximately 5% of protein synthesis in beta‐cells.^47^ Higher demand for insulin synthesis (eg, by 3–5‐fold in obesity and insulin resistance) leads to misfolding of proinsulin. Cells handle misfolded proteins by unfolded protein response,^48^, ^49^ further promoting ER stress, and triggering beta‐cell apoptosis.^47^ These findings have been reported in beta‐cells from donors with T2DM when exposed to moderate hyperglycemic conditions in vitro (~200 mg/dL).^50^ Excessive ER stress subsequently induces apoptosis.^51^, ^52^, ^53^ Additionally, exposure to high levels of both glucose and fatty acids is associated with greater depletion of the normally high ER calcium gradient exacerbating ER stress.^54^, ^55^ The beta‐cell response to misfolded proinsulin is not uniform across all islets as evidenced by nonuniform degrees of insulin production.^32^ This may reflect the known heterogeneous recruitment of beta‐cells in response to glucose‐stimulation.^33^

A less understood proposed mechanism of beta‐cell dysfunction is amyloid deposition. The presence of amyloid deposit in pancreatic islets has been known for many decades^56^ and has been identified as islet amyloid polypeptide (IAPP) or amylin.^57^ Pro‐IAPP is secreted in response to glucose, is cleaved into IAPP and aggregates into β‐pleated sheets. Higher amounts of amyloid are present in islets of patients with poorly controlled T2DM,^58^ and they appear to play a role in beta‐cell dysfunction and death^59^; however, the exact mechanism of possible injury is under investigation.^60^, ^61^, ^62^

There is increasing evidence for a degree of cell plasticity within pancreatic islets. Murine models of T2DM provide evidence of transdifferentiation of beta‐cells into alpha‐cells and delta‐cells, and de‐differentiation into nonsecreting endocrine progenitor cells.^31^, ^60^ This appears to occur predominantly through the loss of transcription factors necessary for beta‐cell maintenance such as FoxO1.^31^ Small, yet rigorous, studies of human donor pancreata from patients with T2DM using lineage tracing of islet cells have shown >3‐fold increase in de‐differentiated cells in those with diabetes compared to controls, as well as suggesting that re‐differentiation from alpha to beta‐cells may also be possible.^61^ The clinical implications of these findings are vast, and the transdifferentiation of beta‐cells into alpha‐cells may in part explain the hyperglucagonemia observed in T2DM.

### 2.3 Methods utilized for measuring beta‐cell function

Beta‐cell function can be defined as the capacity to produce, store, and appropriately release insulin. Importantly, insulin secretory capacity reflects beta‐cell function when it is assessed in the context of prevailing insulin sensitivity.^62^ In normal individuals there is a fixed and predictable hyperbolic relationship between insulin secretion and insulin sensitivity commonly expressed as DI.^63^, ^64^ Hence, it is critical to incorporate insulin sensitivity into the assessment of beta‐cell function.^65^

Various methods are available to assess beta‐cell function and insulin secretory capacity; these methods have been reviewed previously^13^ and are summarized in Table 1. In addition, there is growing interest in using noninvasive modalities to visualize and measure beta‐cell mass.^66^

**TABLE 1:** Various available methods for assessment of beta‐cell function.

Test type with overview	Parameters assessed	Comments/limitations
Static measures assessed under fasting condition		
Insulin and C‐peptide: Fasting sample obtained along with prevailing glucose level.^186^	Insulin and C‐peptide	Not a highly accurate measure; might reflect beta‐cell stress
Proinsulin‐to‐insulin ratio: Fasting sample obtained.^187^	Proinsulin/insulin ratio	Surrogate marker of inappropriate intracellular processing of the pro‐hormone to insulin.
Homeostasis Model Assessment‐β‐cell (HOMA index): HOMA is calculated using steady‐state blood concentrations of fasting glucose and insulin to estimate the degree of beta‐cell function.^188^	HOMA‐β	It has been used in various epidemiological studies.
Measures derived from dynamic testing		
Oral glucose tolerance test (OGTT): After an overnight fast, subjects are given 75‐g oral glucose load. Plasma glucose, C‐peptide, and insulin concentrations are measured at baseline and sequentially over the 120 min.^186^	Insulinogenic index (IGI), Matsuda index, and disposition index (DI)	Relatively easier as compared to other dynamic testing, thus, it is commonly used in experimental settings to evaluate beta‐cell function.
Mixed meal tolerance test (MMTT): After an overnight fast, subjects consume standardized calorie load ingested as a mixed meal within a specified time period. Plasma glucose, C‐peptide, and insulin are measured at baseline and over 2 h (or longer) following meal ingestion.^189^	IGI, Matsuda index, and DI	Though it provides a more physiologically relevant comparison to human meal consumption, the differences in size and composition of the enteral load leads to differences in insulin and incretin responses. Not highly standardized.
Intravenous glucose tolerance test (IVGTT): An IV bolus of dextrose is given and rapid sampling for measurement of glucose and insulin concentrations is performed during the first 10 min (first phase) and subsequently across the remainder of the test to derive the late (second) phase responses.^190^	IGI, DI, Matsuda index, acute insulin release (AIRg).	Can be done faster (10 min) to assess the first phase but usually needs longer time to get all the necessary time points to assess the second phase of insulin secretion. This test also misses the incretin response seen in oral testing.
Hyperglycemic clamps: Plasma glucose concentration is rapidly (within minutes) raised with a large priming IV infusion of glucose. The desired hyperglycemic plateau is maintained by adjusting a variable IV glucose infusion, based on the negative feedback principle. Frequent blood samples are obtained for measurement of glucose, insulin and C‐peptide. In addition, multiple glycemic levels can be achieved, as needed. The technique is sometimes paired with arginine stimulation at the end of the clamp period to measure maximum insulin secretory capacity at a steady‐state glucose concentration.^191^	AIRg, second‐phase insulin response, total amount of endogenously secreted insulin (area under the curve), acute insulin response to arginine (AIRarg) reflecting maximal capacity of insulin secretion.	Though it is a highly sensitive procedure given the complex set up and lack of expertise and availability as well as the cost involved, it is usually used for metabolic research purposes.

### 2.4 Effect of various interventions on beta‐cell preservation

#### 2.4.1 Lifestyle modification

The association between weight loss and the prevention (or delay of onset) of T2DM is well known. The Finnish Diabetes Prevention Study,^67^ the Diabetes Prevention Program,^68^ and their respective long‐term follow‐up studies^69^, ^70^ demonstrated a >30% relative risk reduction of diabetes incidence with weight loss. In individuals with obesity but without T2DM, modest weight loss of ~5% results in significant improvement in insulin sensitivity,^71^ and greater weight loss of ~10% results in significant improvements in beta‐cell function.^72^, ^73^ (Table 2).

**TABLE 2:** Effect of lifestyle modification, hypocaloric diet, and bariatric surgery on beta‐cell function.

Study/references	No. of patients	Age, yrs; male, %; Baseline BMI (kg/m^2^)	DM duration (years); Baseline HbA1c (%)	Intervention and its duration	Washout period prior to assessment of beta‐cell function	Time of assessment	Measures of beta‐cell function	Outcomes
Lifestyle modification								
Magkos et al, 2016^71^	40	44	Nondiabetic	Low calorie diet, 15% average weight loss	n/a	Baseline At time of achievement of weight loss (3.5–10.4 mos)	Total insulin secretory response via OGTT × insulin sensitivity during hyperinsulinemic‐euglycemic clamp	Improved beta‐cell function in weight loss group from a baseline of 6860 to: 5% weight loss: 8130 10% weight loss: 10607 15% weight loss: 11107
		‐						
		37.9						
Utzschneider et al, 2004^73^	19	65.4	Nondiabetic	Low calorie diet, 10% average weight loss	n/a	Baseline 3 months	DI (derived from IV GTT)	DI improved by 33% (9.63–12.78)
		100						
		30.9						
							Insulin sensitivity measures	Insulin sensitivity improved by about 57% (2.65–4.15)
Bariatric surgery compared to low calorie diet								
Hofsø et al, 2011^88^	148	42–51	Nondiabetic, includes IGT group	Gastric bypass versus lifestyle modification	n/a	Baseline One year	DI (derived from OGTT)	Significantly greater improvement in DI in surgery versus lifestyle intervention group
		30						
		43–47						
Yoshino et al, 2020^87^	22	49–54	9	Low calorie diet versus RYGB to target equivalent weight loss	GLP‐1 agonist: 2 wks Oral diabetes agents: 3 days Insulin: 1 day	Baseline Three weeks after target weight loss achieved	DI (derived from mixed meal tolerance test)	DI increased by 1.83 in diet group
		32	7.2–8					
								DI increased by 1.11 in RYBG group
		43						
								No between group differences in rise in DI
Plum et al, 2011^91^	14	50.5	5–8	Low calorie diet versus RYBG; nonrandomized	1–3 days	Baseline Achievement of matched weight loss of 8%	DI (derived from IVGTT)	Non‐significant change in DI in low‐calorie diet group
		35	7.5–7.8%					
		43–46						
								Significant increase in DI in RYGB group
Bariatric surgery								
Kashyap et al., 2010^90^	16	52	5	RYGB versus GR surgery	Diabetes agents: 24 h	Baseline 1 and 4 weeks after surgery	DI (derived from hyperglycemic clamp and MMT)	DI for bypass group unchanged at 4 weeks DI for restrictive surgery group increased by 30% at 4 weeks Matched target weight loss between groups
		56	7.2					
		47						
Plum et al, 2011^91^	17	40–47	Nondiabetic	Gastric banding versus RYGB; Nonrandomized	n/a	Baseline Achievement of matched weight loss of 7.5%	DI (derived from IVGTT)	Significant increase in DI in Gastric Banding group
		11						
		45–48						
								Nonsignificant change in DI in RYGB group
								Note: baseline parameters differed between groups
Kashyap et al, 2013^96^	60	48.4	8.4	IMT versus IMT + RYGB versus IMT + sleeve gastrectomy	Diabetes agents: 24 h	Baseline 12 months 24 months	DI (derived from MMT)	DI increased by 5.8 fold in the gastric bypass group at 24 months from baseline Negligible change in DI in IMT and sleeve gastrectomy groups
		40.7	9					
		36.1						
Hofsø et al, 2019^92^	109	47–48	5	RYGB versus sleeve gastrectomy	Diabetes agents: unknown	Baseline 12 months	DI (derived from IVGTT)	DI increased similarly in both groups
		33	7.9					
		42						Diabetes remission rate higher in RYGB, risk difference 27%
								Weight loss 6% greater in RYGB
Bradley et al, 2014^94^	14	37–40	Nondiabetic	RYGB versus Sleeve gastrectomy	n/a	Baseline Achievement of weight loss target (4–6 mos post‐op)	DI (derived from hyperinsulinemic euglycemic clamp and MMT)	Similar and significant increase in DI in both groups
		21						
								Achieved matched weight loss of around 22%
		50–54						
Mullally et al, 2019^93^	20	44	4	RYGB versus Sleeve gastrectomy	Oral DM agents: 2–3 days	Baseline Achievement of target weight loss achieved (2.5 weeks post‐op)	DI (derived from IVGTT)	Similar and significant increase in DI in both groups
		35	7.4					
		45.2						
								Achieved matched weight loss of around 8.4%

Abbreviations: BMI, body mass index; DI, disposition index; DM, diabetes mellitus; GLP‐1, glucagon‐like peptide‐1; GR, gastric restrictive; HbA1c, glycated hemoglobin; IGT, impaired glucose tolerance; IMT, intensive medical therapy; IVGTT, IV glucose tolerance test; MMT, mixed meal tolerance; OGTT, oral glucose tolerance test; RYGB, Roux‐en‐Y gastric bypass.

In individuals with short duration of T2DM, 10–15 kg weight loss is associated with 46% and 36% glycemic remission rates at 12 and 24 months, respectively, with normalization of insulin secretory capacity.^74^, ^75^ Maintenance of weight loss seems to be an important factor in the sustainability of beta‐cell function improvements over time.^76^, ^77^ Conversely, weight regain is associated with attrition of these improvements.^78^

Remarkably, normalization of insulin secretion can occur after as little as 1 week of a very low calorie diet (VLCD, 600 kcal/day) and is sustained for several weeks after cessation of the intervention.^75^ Longer interventions such as 8 weeks of VLCD achieving ~15 kg weight loss are associated with sustained improvement in insulin secretion several months later,^79^ whereas 6 months of VLCD achieving ~12 kg weight loss has been associated with 60% glycemic remission at 1 year.^80^ Reduction in liver and pancreatic fat content may play an important role in promoting beta‐cell function.^81^ It is important to note that glycemic remission and improvements in beta‐cell function described here were all attained in those with early onset T2DM (<6 years duration) and therefore likely higher beta‐cell mass at the onset of the intervention. Additionally, measurement of beta‐cell indices after a “washout” period at the conclusion of dietary intervention is necessary to assess true improvement in beta‐cell function.

Although this review does not include a comprehensive evaluation of the effects of exercise on beta‐cell function, it is worth noting that improvements in DI have been shown to accompany short‐term interventions including moderate‐intensity training of long duration and high‐intensity interval training^82^, ^83^, ^84^ with improvements correlating strongly with abdominal fat loss.^84^ Whether improvements in beta‐cell function are sustained after these interventions are concluded requires further exploration.

#### 2.4.2 Bariatric surgery

The mechanisms of beta‐cell improvement following bariatric surgery are to some extent similar to those with VLCD.^75^, ^85^ The degree of weight loss is thought to be the major determinant of beta‐cell recovery regardless of the type of surgery performed.^86^, ^87^, ^88^ The entero‐insular response, particularly higher GLP‐1 and gastric inhibitory polypeptide (GIP) blood levels, has been proposed as a mediator of improvement independent of the degree of weight loss.^89^, ^90^, ^91^ Some studies suggest that bariatric surgery is more effective than low calorie diets (LCD) for the treatment (and reversal) of T2DM, but a valid comparison of these interventions is challenging and have reached different conclusions. Yoshino et al reported no statistical differences in the degree of improvements in insulin sensitivity and beta‐cell function between the gastric bypass surgery (GB) and diet groups, both having attained an average weight loss of 17%–18% of body weight.^87^ Plum et al reported significantly higher improvements in insulin secretion and DI following GB compared to LCD with matched weight loss and as early as 3 weeks after surgery (Table 2).^91^

The two most commonly performed metabolic surgeries—Roux‐en‐Y GB (RYGB) and sleeve gastrectomy—appear to have similarly favorable effects on beta‐cell function,^92^, ^93^, ^94^ although other reports show superiority of the Roux‐en‐Y.^95^, ^96^ The positive effects appear to be sustained over time.^92^ Importantly, similar to interventions with LCDs, large improvements in beta‐cell function post surgery have predominantly involved patients with shorter duration of T2DM and therefore likely higher beta‐cell reserves. While this manuscript was under preparation and review, a study detailing the long‐terms effects of RYGB was published.^97^ The study showed durable improvements in beta‐cell function at 2 years following surgery, with presurgical beta‐cell functional status being an important predictor of these improvements rather than the degree of weight loss or improvements in insulin sensitivity.

#### 2.4.3 Insulin therapy

A short duration of intensive insulin therapy (IIT) has favorable effects on insulin secretory capacity.^98^, ^99^, ^100^, ^101^, ^102^, ^103^, ^104^, ^105^ The reduction in demand for endogenous insulin is thought to provide “beta‐cell rest,” diminishing beta‐cell apoptosis and possibly improving beta‐cell function. Additionally, binding of insulin to its receptors on beta‐cells is purported to improve beta‐cell function.^106^ Beta‐cells driven to de‐differentiation by prolonged hyperglycemic conditions may be recruited for insulin synthesis following beta‐cell rest and improved glycemia afforded by IIT (Table 3).^107^

**TABLE 3:** Effect of insulin therapy on beta‐cell function.

Study/references	No. of patients	Age, yrs; male, %; Baseline BMI (kg/m2)	DM duration (years); baseline HbA1c (%)	Intervention and its duration	Washout period prior to assessment of beta‐cell function	Time of assessment	Measures of beta‐cell function	Outcomes
Hu et al., 2011^108^	48	50.6	New diagnosis	2 weeks of IIT with basal/bolus insulin	• > 10 months in remission group. • Unknown in non‐remission group	Baseline End of 2 weeks of IIT 12 months of follow up	DI (derived from 25 gm IVGTT)	Remission group: DI improved from 11.7 (7.3–15.9) to 41.2 (30.8–80.8) at 1 year Non‐Remission group: DI improved from12.4 (9.2–16.2) to 21.3 (18.2–37.3) at 1 year.
		70						
		25.7	10 ± 2.2					
Kramer et al., 2013^110^	63	59 ± 8.2	3 ± 2.1 yrs	4 weeks of IIT with basal/bolus insulin	1 day	Baseline End of 4 weeks of IIT	ISSI‐2 (derived from OGTT)	ISSI‐2 increased by a mean of 25%
		63	6.8 ± 0.8					
		N/A						
Xu et al., 2015^111^	342	51	new diagnosis	48 weeks of pre‐mix insulin	2 days	Baseline End of 48 weeks of IIT	DI (derived from MMT)	DI increased from 0.6 to 1.1 at 48 weeks
		61	8 ± 0.1					
		25.9						
Bunck et al., 011^113^	69	58.3	4.0 ± 0.6 yrs	3 years of glargine + metformin	4 weeks	Baseline 4 weeks after 3 years of IIT	DI (derived from combined euglycemic and hyperglycemic clamp)	DI declined by 0.99 ± 0.65
		65.3	7.4 ± 0.1					
		30.1						
RISE Consortium, 2018^115^	91	14.4	59% IGT	3 mos of glargine followed by 9 mos of metformin	12 weeks off insulin therapy and >12 h after last metformin dose	Baseline End of 52 weeks of therapy 12 weeks off therapy	2 hyperglycemic clamp‐derived beta‐cell responses paired with insulin sensitivity	Both measures showed significant decline at 15 weeks compared to baseline.
		28.6	41% T2DM of <6 mos duration					
		36.7	5.7 ± 0.6					

Abbreviations: DI, disposition index; IIT, intensive insulin therapy; ISSI‐2, insulin sensitivity‐secretion index‐2; IGT, impaired glucose tolerance; IVGTT, IV Glucose Tolerance test; OGTT, oral glucose tolerance test.

Hu et al studied the effect of 2 weeks of IIT in newly diagnosed T2DM.^108^ Remission rate of hyperglycemia was 44% at 1 year accompanied by significant improvements in insulin sensitivity though only modest improvement of DI. Kramer et al^109^ studied the effect of 4 weeks of IIT T2DM of <7 years’ duration. Only one third of the patients showed significant improvement in beta‐cell function, and only patients with improvement in homeostatic model assessment of insulin resistance (HOMA‐IR) manifested improved function. Improvement in insulin sensitivity has been observed using short‐term IIT and may be the main driver of clinical outcomes.^110^ Factors that correlated with improvement in beta‐cell function and a longer duration of remission included short duration of T2DM, better glycemic control, and higher insulin sensitivity at baseline.^110^

The effect of longer durations of insulin therapy on beta‐cell function has also been examined. Xu et al reported a significant improvement in beta‐cell function in patients with newly diagnosed T2DM after 48 weeks of insulin therapy.^111^ In patients with 4‐year average duration of T2DM, 52 weeks of insulin therapy resulted in only transient improvements in insulin secretion.^112^ Extension of therapy to 3 years in this cohort demonstrated a reduction in beta‐cell function, perhaps reflecting the natural decrease of beta‐cell function over time.^113^ In the large Outcome Reduction With Initial Glargine Intervention (ORIGIN) Trial, prolonged use of glargine insulin of up to 6 years significantly reduced the progression from impaired glucose tolerance to T2DM.^114^ Although rigorous measures of beta‐cell function were not performed, it is important to note that this benefit occurred despite some weight gain with insulin therapy.

At the present time, it seems unclear whether acute or chronic insulin therapy produces true improvement of beta‐cell function in T2DM. Alleviation of glucolipotoxicity might be the predominant pathway to any observed improvements. Of particular concern is the poor response of youth with T2DM to IIT, where a continual decline in beta‐cell function is seen despite insulin and metformin therapy.^115^

#### 2.4.4 DPP‐4 inhibitors

Dipeptidyl peptidase 4 (DPP‐4) inhibitors hinder the degradation of endogenous GLP‐1 and result in a modest (~2‐fold) rise in its blood levels.^116^ Although numerous trials have investigated the effects of this class of medications on beta‐cell function, most did not include a “washout” period or rigorous measures such as DI. Improvements in HOMA of β‐cell function (HOMA‐β) and HOMA‐IR was demonstrated in multiple trials at the conclusion of a 24‐week course of DPP‐IV inhibitor therapy prior to washout.^117^, ^118^ The combination of metformin and vildagliptin in drug‐naïve patients with T2DM for 12 months led to a decline in both HOMA‐IR and glucagon levels, as well as an increase of HOMA‐β compared with metformin alone while on therapy.^119^ Trials that have employed “washout” have consistently shown loss of benefits on beta‐cell function after cessation of therapy.^120^, ^121^, ^122^ In sum, DPP‐IV inhibitors can lead to some enhancement of beta‐cell secretory capacity in patients with prediabetes and in drug‐naïve patients with T2DM; however, these benefits are not sustained after termination of therapy and cannot be construed as enhancement in beta‐cell function (Table 4).

**TABLE 4:** Effect of Incretin‐based therapies on beta‐cell function.

Study/References	No. of patients	Age, yrs; male, %; baseline BMI (kg/m2)	DM duration (years); baseline HbA1c (%)	Intervention and its duration	Washout period prior to assessment of beta‐cell function	Time of assessment	Measures of beta‐cell function
DPP‐4 Inhibitors							
Utzschneider et al, 2008^120^	22	59.6	IFG 6	Vildagliptin for 6 weeks	2 weeks	Baseline 6 weeks 2 weeks after wash out	DI (derived from frequently sampled intravenous glucose tolerance test) (AIRg x SI)
		50					
		29.7					
D'Alessio et al, 2009^121^	41	55	3.5 6.7	Vildagliptin for 3 months	2 weeks	Baseline 12 weeks 2 weeks after wash out	DI (derived from intravenous glucose tolerance test) (AIRg x SI)
		32.4					
Scherbaum et al, 2008^122^	131	63.1	2.3 6.6	Vildagliptin for 56 weeks, then 4 weeks washout, followed by 56 weeks extension	4 weeks	Baseline 24 weeks 52 weeks 80 weeks 108 weeks 4 weeks after washout	Beta cell function measured by MMT (ISR/G). ISR‐insulin secretory rate (AUC_0–2 h_). G‐glucose area under the curve (AUC_0–2 h_)
		39					
		30.3					
GLP‐1 receptor agonists							
Bunck et al, 2011^113^	69	58.3	4.9	3 years of exenatide + metformin	4 weeks	Baseline 52 weeks 168 weeks • 4 weeks after wash out	DI (derived from combined euglycemic and hyperglycemic clamp)
		65.3	7.5				
		30.5					
Retnakaran et al, 2014^135^	51	58.2	2.25	Prerandomization 4 weeks of IIT followed by liraglutide for 48 weeks	2 weeks	Baseline 12, 24, 36 and 48 weeks 2 weeks after washout	ISSI‐2 (derived from 75 gm OGTT)
		62.8	30.2				
			6.3				
RISE Consortium, 2019^136^	267	53.9	IGT or newly diagnosed with diabetes 5.75	liraglutide and metformin for 12 months	3 months	Baseline 12 months 3 months after wash out	Hyperglycemic clamp‐derived C‐peptide response measures paired with concurrently measured insulin sensitivity
		57.2					
		35					

Abbreviations: AIRg, acute insulin response to glucose; AUC, area under curve; BMI, body mass index; DI, disposition index; DM, diabetes mellitus DPP‐4, dipeptidyl peptidase 4; GLP‐1, glucagon‐like peptide‐1; HbA1c, glycated hemoglobin; IFG, impaired fasting glucose; IGT, impaired glucose tolerance; IIT, intensive insulin therapy; ISSI‐2, insulin secretion‐sensitivity index‐2; MMT, mixed meal tolerance; OGTT, oral glucose tolerance test; RISE, Restoring Insulin Secretion; SI, insulin sensitivity.

#### 2.4.5 GLP‐1 receptor agonists

GLP‐1 RAs not only improve hyperglycemia but also cause significant weight loss, are cardioprotective, and appear to have some protective effects against kidney disease and fatty liver disease.^123^, ^124^, ^125^, ^126^ Data in animal models have suggested that GLP‐1 RAs may induce islet‐cell neogenesis, inhibit beta‐cell apoptosis, and increase beta‐cell mass (Table 4).^127^, ^128^

In 2004, Degn et al reported that a short (1‐week) treatment with liraglutide improved proinsulin‐to‐insulin ratio, HOMA‐B, first‐phase insulin response to glucose, and DI.^129^ Mari et al, Buse et al, and DeFronzo et al studied the effect of exenatide in patients on sulfonylureas ± metformin. They found that exenatide increased insulin secretion rates by 40%–72% after 30 weeks of treatment.^130^ Buse et al and Defronzo et al reported a significant decrease in the proinsulin‐to‐insulin ratio in the group treated with exenatide for 30 weeks.^131^, ^132^ Multiple studies evaluating the effect of treatment with liraglutide (ranging from 2 to 48 weeks) have reported significant improvement in beta‐cell function.^133^, ^134^ It is important to note that these studies assessed beta‐cell function without a “washout” period.

Studies investigating the effect of GLP‐1 RAs on beta‐cell function after a “washout” period are outlined in Table 4. Bunck et al showed that exenatide compared to insulin glargine improved pancreatic beta‐cell secretory function against a background of similar glycemic control over a 1‐year period; however, these findings were not sustained after a 4‐week “washout” period.^112^ Of note, some patients in this cohort were continued on exenatide or glargine treatment for a total of 3 years. The group treated with exenatide demonstrated a sustained improvement in DI, and this change was sustained after 4 weeks of washout whereas a reduction in DI was observed in the group treated with glargine.^113^ Additionally, exenatide treatment was associated with continued weight loss and improvement in whole body insulin sensitivity.^113^ The Liraglutide and Beta‐cell Repair (LIBRA) trial evaluated the effect of liraglutide on beta‐cell function in individuals with T2DM and found beneficial effects after 48 weeks of treatment; however, the benefits were lost after 2 weeks of “washout.”^135^ Similarly, in the Restoring Insulin Secretion (RISE) Consortium trial the large on‐treatment positive effect of liraglutide on beta‐cell function at 12 months in participants with either impaired glucose tolerance (IGT) or recently diagnosed T2DM disappeared when assessed 3 months after treatment withdrawal.^136^ Le Roux et al showed that the larger dose of liraglutide (3 mg daily) used for 3 years in patients with prediabetes was associated with an improvement in beta‐cell function.^137^ After 3 months of withdrawal from treatment, only the effects on fasting insulin and HOMA‐IR were sustained.

In sum, GLP‐1 RAs lead to significant improvement of beta‐cell function *while* on continuous treatment. There is at least some evidence that a longer duration of treatment (perhaps 3 years or more) could potentially result in long‐lasting alteration of beta‐cell function. We look forward to further long‐term data on GLP‐1RA use to support or repudiate this. Additional factors including duration of T2DM, glycemic control, and body weight reduction may play critical roles in the ultimate efficacy of the GLP‐1 RAs to improve beta‐cell function.

#### 2.4.6 Sodium‐glucose cotransporter‐2 inhibitors

Sodium‐glucose cotransporter‐2 (SGLT2) transporters facilitate sodium and glucose reabsorption in renal proximal tubules and mediate ~90% of renal tubular reabsorption of glucose.^138^ Although SGLT2i(s) do not appear to have a direct effect on beta‐cells, the decrease in glucose toxicity and the increase in pancreatic lipid oxidation lead to an increase in response of beta‐cells to glucose,^75^, ^139^, ^140^, ^141^ an augmented response to GLP‐1, and an enhanced first phase of insulin secretion.^142^ Other proposed positive effects include upregulation of musculoaponeurotic fibrosarcoma oncogene homolog A (MafA) and pancreatic and duodenal homeobox 1 (PDx‐1) transcription factors involved in insulin biosynthesis.^143^ Preclinical studies in mice with deletion of SGLT2 gene showed improvement of beta‐cell function.^144^

There is evidence of rapid effect of SGLT2i(s) on beta‐cell function as early as 48 h^139^, ^140^, ^145^ with improvement of insulin secretion, insulin sensitivity, and DI.^139^, ^140^, ^145^, ^146^ Long‐term use of SGLT2i(s) showed improved sensitivity of beta‐cells to glucose while on treatment.^147^, ^148^

Takahara et al reported two separate studies showing improvements in glucose levels, insulin secretion and DI with 4 weeks of treatment with ipragliflozin and 24 weeks of treatment with canagliflozin; benefits were sustained after a 1‐week “washout” period^149^, ^150^ It may be worth noting that the average duration of diabetes in the latter study was more than 10 years.^150^ At present, it is difficult to definitively conclude that there is a positive effect of SGLT2i(s) on beta‐cell function.

#### 2.4.7 Metformin

Metformin decreases hepatic glucose production leading to lower blood glucose and insulin levels and a small degree of weight loss. These changes likely contribute to the small increase in insulin sensitivity.^151^, ^152^, ^153^ Additional favorable effects include attenuation of lipotoxic effects^154^, ^155^ and decreases in rates of beta‐cell apoptosis.^156^ Nonetheless, these effects do not result in preservation of beta‐cell function over time. In the Diabetes Prevention Trial, metformin improved insulin sensitivity and reduced progression to T2DM by 31%,^68^ a smaller effect compared to the lifestyle group; however, these benefits diminished significantly after a “washout” period.^157^ In the A Diabetes Outcome Progression (ADOPT) trial, improvements in insulin sensitivity and secretion in patients with T2DM treated with metformin diminished significantly after 6 months off treatment.^158^ The RISE Consortium showed similar transient beta‐cell benefits using 12 or 24 months of metformin in obese patients with prediabetes or recent onset of T2DM, with most improvements lost by the end of therapy.^136^, ^159^ Finally, metformin was less effective in controlling glycemia in youth with recent onset T2DM compared to adults^160^ with a progressive decrease of DI despite continued therapy.^161^

#### 2.4.8 Thiazolidinediones

Thiazolidinediones (TZDs) are peroxisome proliferator‐activated receptor‐gamma agonists that regulate transcription of genes involved in lipid and carbohydrate metabolism; they are expressed in various tissues including pancreatic beta‐cells.^162^ Beneficial effects of TZDs on beta‐cell function include the prevention of FFA‐induced downregulation of insulin gene expression and restoration of glucose‐stimulated insulin release,^163^ as well as the upregulation of GLUT2 and glucokinase expression involved in “glucose‐sensing” by beta‐cells.^164^

Studies have shown that TZDs delay the progression from prediabetes to T2DM. For example, troglitazone enhanced insulin sensitivity and improved glucose tolerance in individuals with IGT.^165^ In patients with gestational diabetes mellitus, both troglitazone and pioglitazone improved acute insulin response to glucose and delayed the progression to T2DM.^166^, ^167^ In the Actos Now for Prevention of Diabetes (ACT NOW) study, pioglitazone decreased the conversion of IGT to T2DM by 72% and resulted in an increase in DI.^168^ In the Diabetes Reduction Assessment with ramipril and rosiglitazone Medication (DREAM) study, treatment with rosiglitazone over 3 years reduced the conversion from IGT to T2DM by 62%,^169^ but these benefits were not sustained off therapy.^170^ In summary, although TZDs are highly effective in preventing the progression from prediabetes to T2DM, their positive effects on beta‐cell function do not persist off therapy. As such, continuous treatment with TZDs may preserve, but not enhance, beta‐cell function.

#### 2.4.9 Sulfonylureas

The negative effects of sulfonylureas on beta‐cell function became evident in the UK Prospective Diabetes Study (UKPDS) trial^171^ and were further substantiated in the ADOPT trial.^158^ The loss of beta‐cell function was characterized by a progressive decline in insulinogenic index and insulin sensitivity.^158^ Gliclazide appears to have unique antiapoptotic properties demonstrated in vitro^172^, ^173^ and lower glycemic failure rates compared to other sulfonylureas,^174^ but rigorous studies of its effects on beta‐cell function are lacking. While our study was under review, the Glycemia Reduction Approaches in Diabetes (GRADE) study showed that use of the sulfonylurea glimepiride resulted in good glycemic control, implying that there was no major loss of beta‐cell function over a period of 5 years.^175^ However, the details of beta‐cell function that were measured during the GRADE study are currently under analysis.

#### 2.4.10 Therapy with a combination of agents

There have been some studies examining beta‐cell function using a combination of glycemia‐lowering agents. Most of these studies have not included a “washout” period. Nevertheless, some of them are briefly discussed here because they address whether the effect of combination therapy might be additive or perhaps synergistic. DeFronzo et al examined the effect of a 20‐week therapy with exenatide or rosiglitazone and their combination on beta‐cell function; there was a significant increase in DI using exenatide alone and exenatide plus rosiglitazone with no difference between the two groups.^176^ The effect of 16 weeks of therapy with either saxagliptin or dapagliflozin and their combination (on a background of metformin therapy) on C‐peptide/insulin ratio was examined by Ekholm et al; they found a similar positive effect using either dapagliflozin alone or the combination.^177^ Likewise, Ali et al examined the effect of canagliflozin, liraglutide, and their combination on beta‐cell function^148^; they reported positive effects on both beta‐cell function and beta‐cell sensitivity to glucose using liraglutide alone or the combination without a difference between the two groups, suggesting that the observed effect was primarily due to liraglutide. More studies are needed to examine the effect of combination therapy on beta‐cell function.

## 3 CONCLUSIONS AND FUTURE DIRECTIONS

As summarized in this review, results of studies in the past decade have shown that enhancement of beta‐cell function is indeed feasible. Enhanced beta‐cell function is a profound positive outcome of sustained weight loss following bariatric surgery or by dietary means. In addition, increased understanding of the pathophysiology of glucose homeostasis facilitated the discovery of two novel classes of medications, namely the GLP‐1 RA(s) and SGLT2i(s). This has led to a paradigm shift in the management of patients with T2DM from a major focus on control of hyperglycemia per se to medications that control hyperglycemia while also decreasing cardiovascular and renal outcomes.^178^, ^179^ Of note, most of the studies examining preservation and enhancement of beta‐cell function have understandably focused on the effects of glycemia‐lowering agents that are used in the treatment of T2DM. Studies exploring use of non‐glycemia‐lowering agents may prove highly fruitful in this endeavor and should be encouraged.^34^, ^35^, ^36^

Given the positive effects of significant weight loss on preservation and enhancement of beta‐cell function, it is likely that more research on use of weight‐lowering therapeutics and dietary measures such as intermittent energy restriction, especially during prediabetes may prove highly effective.^180^, ^181^ A key outcome with use of GLP‐1 RA(s) is notable weight loss, and these agents are becoming cornerstone medications for the treatment of obesity.^124^ Interestingly, the dual‐based GIP/GLP‐1 agonists are under further study for joint treatment of T2DM and obesity.^182^, ^183^, ^184^ Recently, tirzepatide, the newly approved dual GIP/GLP‐1 receptor agonist was found to be superior to semaglutide (selective GLP‐1 RA) with respect to reduction of glycated hemoglobin (HbA1c) and weight loss after 40 weeks of treatment.^185^

Further research is needed to understand the basic mechanisms underlying the reversal of the otherwise progressive loss of beta‐cell function in patients with T2DM. Additionally, identification and use of therapeutic agents (including agents that do not lower blood glucose per se) either singly or in combination that result in enhancement of beta‐cell function are potentially highly productive areas for future research.

## FUNDING INFORMATION

No funding received.

## DISCLOSURE

The authors declare no conflicts of interest.

## Data Availability

The data that support the findings of this study are openly available through the National Library of Medicine.
